# Uses of Borax

**Published:** 1886-10

**Authors:** 


### *USES OF BORAX.*

A cup of powdered borax on your wash-stand will do wonders in the way of softening the skin. If you have been working in the garden, or doing anything about the house which has tended to make your hands rough, when you wash them dip your fingers in the borax and rub your hands well with it. Borax (pulverized) sprinkled plentifully around the haunts of water bugs will drive them away. Cockroaches also will yield to this treatment and depart. The safest and best thing also for washing the hair is a moderately strong solution of borax in water. Pure water should be used immediately after washing with the borax and water.

The washerwomen of Holland and Belgium, who get up their linen so beautifully white, use refined borax as washing powder instead of soda, in the proportion of one large handful of borax powder to about ten gallons of boiling water. They thus save in soap nearly half. All the washing establishments adopt the same mode. For laces, cambrics, etc., an extra quantity of the powder is used, and for crinolines, (requiring to be made stiff,) a strong solution is necessary. Borax being a neutral salt, does not in the slightest degree injure the texture of the linen; its effect is to soften the hardest water, and, therefore, it should be kept on every toilet table. To the taste it is rather sweet; but not at all unpleasant, is an excellent dentifrice, and in hot countries is employed in combination with tartaric acid and bi-carbonate of soda as a cooling beverage Good tea cannot be made with hard water; but all water may be made soft by adding a teaspoonful of borax powder to an ordinary sized kettle of water, in which it should boil. The saving in the quantity of tea used will be at least one-fifth. Our lady readers who have not used borax have been losing a great help and comfort. If once tested, none will be without it on the toilet table. It removes stains and dirt from the hands better than soap, and at the same time softens and smooths the skin. It is excellent for washing laces, and will, without injury, cleanse brushes and combs in a few moments. It extracts dirt from articles of delicate texture without rubbing, it being only necessary to put them to soak in a solution of borax over night, and to rinse them in the morning. Two tablespoonfuls of pulverized borax dissolved in a quart of water, to which add enough water to cover a pair of blankets, will cleanse them beautifully. It also saves great labor in washing paint.

